# Evaluation of the contour of edentulous jaw sections in the transversal plane and the buccolingual vertical-level disparity in CBCT and panoramic radiography images: a retrospective comparative study

**DOI:** 10.1186/s40729-022-00466-8

**Published:** 2023-01-03

**Authors:** Ali Reza Ketabi, Andree Piwowarczyk, Matthias Christian Schulz, Hans-Christoph Lauer, Stefan Hassfeld

**Affiliations:** 1 Department of Prosthodontics, School of Dentistry, Faculty of Health, Witten/Herdecke University, Alfred-Herrhausen-Straße 45, 58455 Witten, Germany; 2 Private Dental Office, Epplestraße 29 a, 70597 Stuttgart, Germany; 3 Department of Oral and Maxillofacial Surgery, University Hospital Tübingen, Eberhard Karls Universität Tübingen, Osianderstraße 2-8, 72076 Tübingen, Germany; 4 Department of Prosthodontics, Center for Dentistry and Oral Medicine (Carolinum), Goethe-University, Theodor-Stern-Kai 7, 60596 Frankfurt, Germany; 5 Department of Oral and Maxillofacial Surgery, Dortmund Hospital GmbH and Faculty of Health, Witten/Herdecke University, Muensterstr. 240, 44145 Dortmund, Germany

**Keywords:** Cone-beam computed tomography, Dental implant, Submandibular fossa, Diagnosis/clinical assessment, Alveolar ridge contour, Available bone width, Lingual undercuts

## Abstract

**Purpose:**

This study investigates whether edentulous jaw sections in the planned implant position exhibit jaw contours funnel-shaped or exhibit pronounced retraction of the jaw (unusual jaw contours) in the transversal plane of the three-dimensional (3D) images, not visible in two-dimensional (2D) images.

**Methods:**

A total of 335 patients with an edentulous section of the jaw that required dental implants were selected. Anonymised radiologic patients’ data were collected, comprising cone-beam computed tomography (CBCT) images of the edentulous jaw sections. In the first stage, unusual jaw contours were examined, including funnel-shaped or pronounced retraction of the jaw and hypodense regions with an undercut and/or bone deficit. In the second stage, the variation in the height of the alveolar ridge between the lingual and buccal contour in the edentulous jaw sections was assessed.

**Results:**

The CBCT images of an unusual jaw contour were observed in 8 cases (2.4%) in the maxilla on the left and 10 cases (3%) in the maxilla on the right. In the mandible, a jaw contour deviates in 39 cases (12.1%) on the left side and 39 cases (12.1%) on the right side. A height difference was detected in the upper jaw in 307 cases and the lower jaw in 265 cases. The discrepancy was 2.09 mm (± 2.25 mm) in the maxilla and 3.97 mm (± 3.45 mm) in the mandible.

**Conclusions:**

The CBCT scan provides useful information to avoid complications in the preoperative planning phase and surgical planning in implant dentistry.

**Graphical Abstract:**

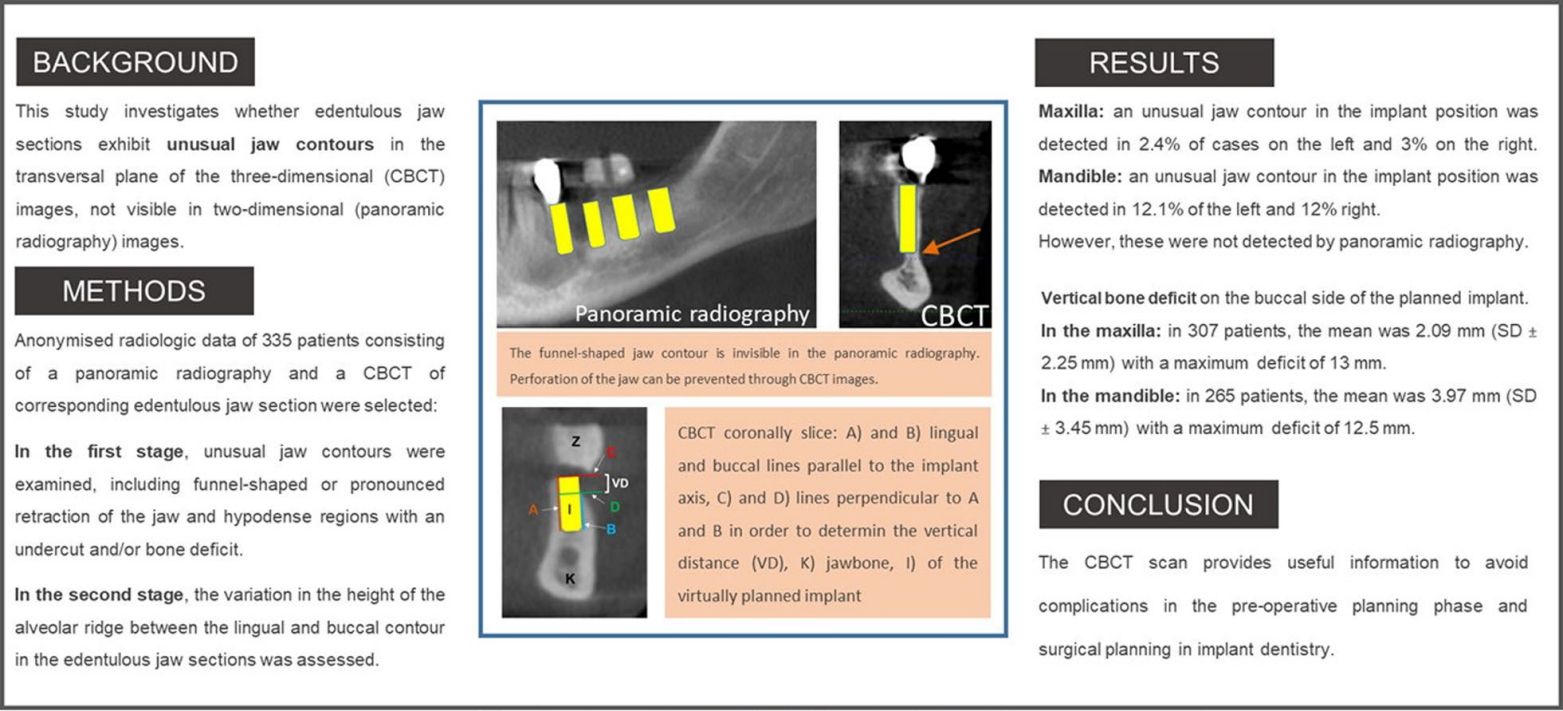

## Background

A profound knowledge of the precise anatomy of edentulous jaw sections and adjacent vulnerable structures is a key factor for successful dental surgery and implant dentistry. It is a crucial factor considering surgical and prosthetic aspects, e.g. when determining possible necessities for augmentation and assessing potential surgical risks [1].

According to a pioneering study by Mozzo et al., three-dimensional (3D) radiographic imaging by cone-beam computed tomography (CBCT) shows obvious benefits for various indications to a pioneering study by Mozzo et al. [2] in 1998. Those indications range across various specialisations the different sections of dentistry, e.g. orthodontics [3–5], endodontics [6, 7], periodontology [8], oral and maxillofacial surgery [9, 10], and implant dentistry [11, 12].

Studies have revealed that CBCT images facilitate accurate representation and precise measurements of anatomical structures [11–13]. Compared with conventional two-dimensional (2D) imaging using panoramic radiography, CBCT offers a distinct advantage in the overlay-free representation of anatomical structures [14].

While some studies have identified no advantages in using CBCT for surgical procedures [9, 10, 15], others advocate its routine use owing to its additional spatial information [16]. Many studies have identified the comparative superiority of CBCT over panoramic radiography for detecting anatomical structures and for planning the insertion of dental implants in the mandible [12, 17, 18] and the maxilla [19, 20]. Owing to its unique advantages, dental practitioners consider CBCT as an essential tool in performing the preoperative phase of implant surgery, identifying potential bone augmentations and in avoiding perioperative complications [21–23].

The current study investigates whether edentulous jaw sections in the planned implant position exhibit jaw contours with bone deficits, such as funnel-shaped or pronounced retraction of the jaw (unusual jaw contours), in the transversal plane of the 3D images not visible in 2D images. The study also measured the difference between the buccolingual height of the alveolar ridge of the intended implant position and direction in the edentulous jaw sections. The hypothesis is that no difference is observed when assessing the contour in edentulous jaw sections or when measuring the buccolingual height in 2D and 3D images.

## Methods

Prior to the start of the study, the approval was obtained from the Ethical Committee of the Baden-Württemberg Medical Association (File reference: F-2014-006-z).

In this retrospective study, radiologic data consisting of a panoramic radiography and a CBCT of corresponding edentulous jaw sections were collected from the patients of a private practice in Stuttgart between 2010 and 2017. The indications for the radiographs were independent from the study.

The inclusion criteria for the study were set up as follows:

- radiologic images depicting corresponding edentulous jaw sections in both panoramic radiography and CBCT images.
- the interval between panoramic radiography and CBCT images was less than 6 months.
- no artefacts in the analysed area.

All images were taken by an expert in dental radiography. Prior to the start of the study, the examiner with the required qualifications and competence in CBCT had received a special training from an expert in dental radiology. Multiple assessments were performed on 20 randomly selected samples to verify the reliability of the radiographic measurements and evaluations. Reliability analysis was conducted after the radiographic instructions and under standardised conditions in a darkened room (< 1000 lx), using an accredited diagnostic monitor (EIZO FlexScan S2000 1024 × 1280 pixels). Measurements were taken over a maximum of 6 h per day and a 30-min interval every 2 h. Inter-rater reliability of the outcomes between the examiner and expert was established. All images were examined a second time by the same examiner, following an interval of 2 weeks for calculating intra-rater reliability. A kappa coefficient was for intra-observer reliability and inter-observer reliability.

Panoramic radiography was recorded using the Orthophos D 3297 X-ray unit (Sirona dental Systems GmbH, Bensheim, Germany) and was saved on an imaging plate (Vistascan View, Dürr Dental, Bietigheim-Bissingen, Germany). The exposure parameters were set up at a tube voltage of 60 kV, at a current of 10 mA and an exposure time of 16.4 s, read out with an imaging plate scanner (Vistascan Combi Plus, Dürr Dental). The evaluation was conducted using the radiographic software DBSWin (version 5.1.1; Dürr Dental SE, Bietigheim-Bissingen, Germany).

The CBCT images were recorded using a Gendex CBX-500™ (KaVo Dental GmbH, Biberach, Germany). The acquisition parameters were a tube voltage of 90 kV, and an exposure time of 8.9 s with a 0.3-mm resolution, using a field of view of 6 or 14 cm (diameter) and 5–8.5 cm (height). The images were evaluated using the i-CAT Viewer software (Imaging Sciences International, Hatfield, PA, USA).

For confidentiality, the patient`s radiologic data were anonymised, and the radiographic images were numbered. MS Excel 2016 chart (Microsoft Inc., Redmond, Washington, USA) was used to process the patient’s data and the radiographic indications.

**Fig. 1:**
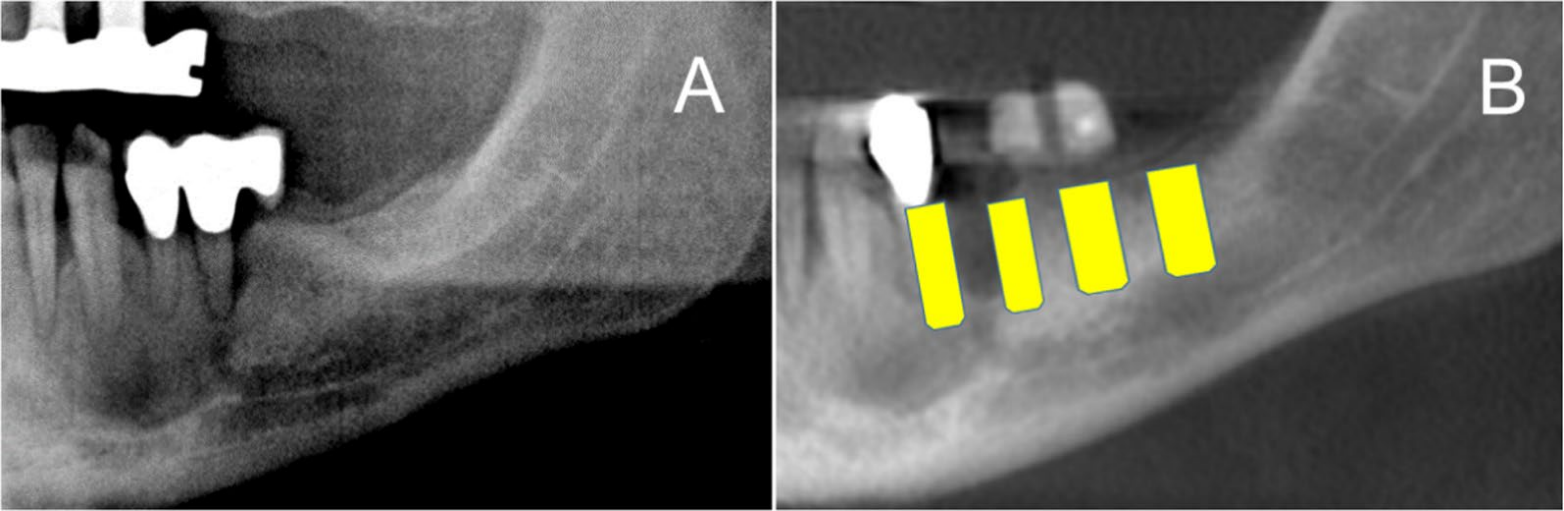
**A** Initial panoramic radiography of the left mandible; **B** the determination of the planned implants in regions 34–37 using an orientation template

**Fig. 2:**
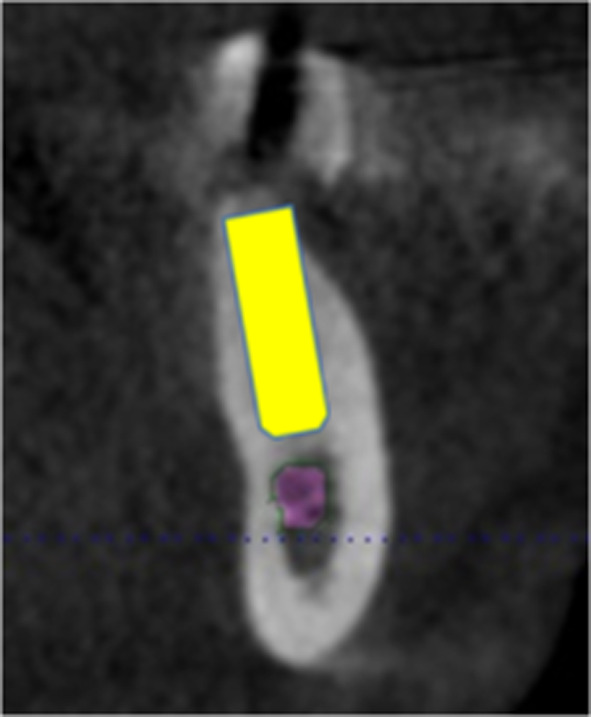
The same patient’s (Fig. 1) CBCT with the virtually planned implant in region 37 showed inconspicuous jaw contour

**Fig. 3:**
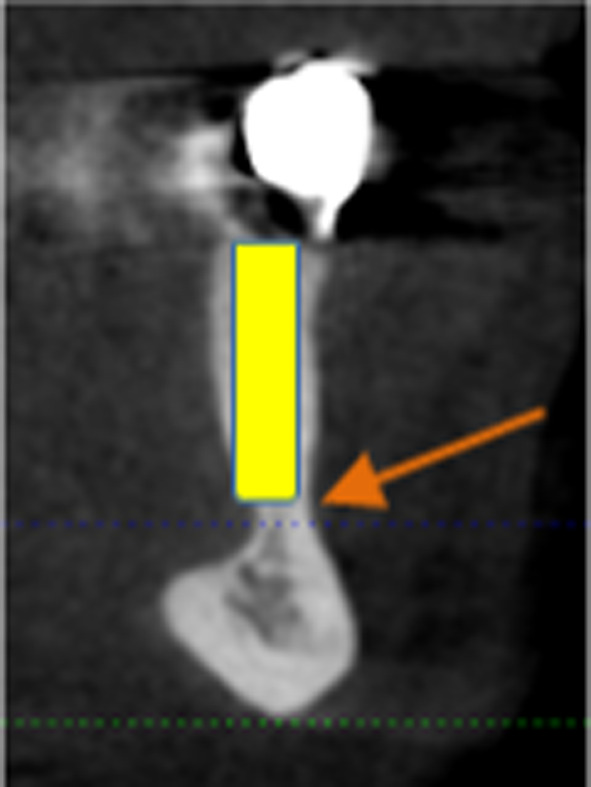
CBCT with virtually planned implant planning in region 35 showing a funnel-shaped contour of the jaw and a pronounced retraction of the jaw contour from the lingual side. The funnel-shaped jaw contour is invisible in the panoramic radiography. Perforation of the jaw can be prevented through CBCT images

The panoramic radiography images (Fig. 1) and the CBCT images were evaluated to detect unusual jaw contours in the planned, prosthetically oriented implant position and direction (Figs. 2, 3). Here, the critical factor was whether a perforation could be detected with a CBCT image during the implant placement as opposed with the implanted placement based on the panoramic radiography image.

The initial step was to diagnose the edentulous jaw sections as revealed in panoramic radiography images. This was followed by the diagnostic analysis of the corresponding regions in the CBCT image.

**Fig. 4:**
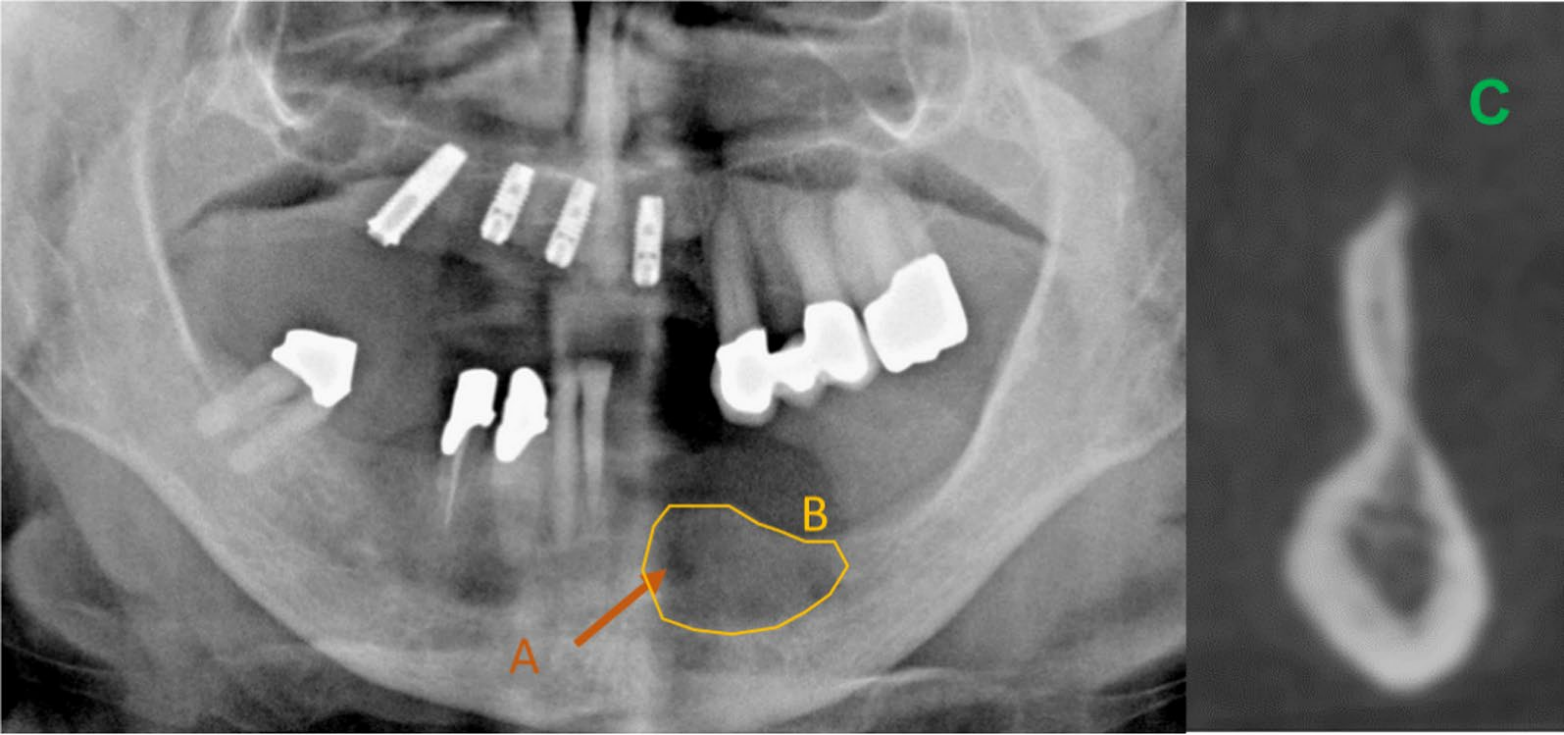
Panoramic radiography image showing A a distinct hypodense lesion and B a translucent area. The depiction of the same region in CBCT (C)

In the case of the panoramic radiography images, the edentulous jaw sections were examined in a vertical direction from occlusal to apical. Hypodense regions were considered evidence of the presence of an undercut and/or bone deficit (Fig. 4). In the case of the CBCT images, unusual contours of the edentulous jaw sections and the presence of any bone deficits were assessed, such as funnel-shaped or pronounced retraction in the vertical direction in the transversal plane (Figs. 2, 3).

**Fig. 5:**
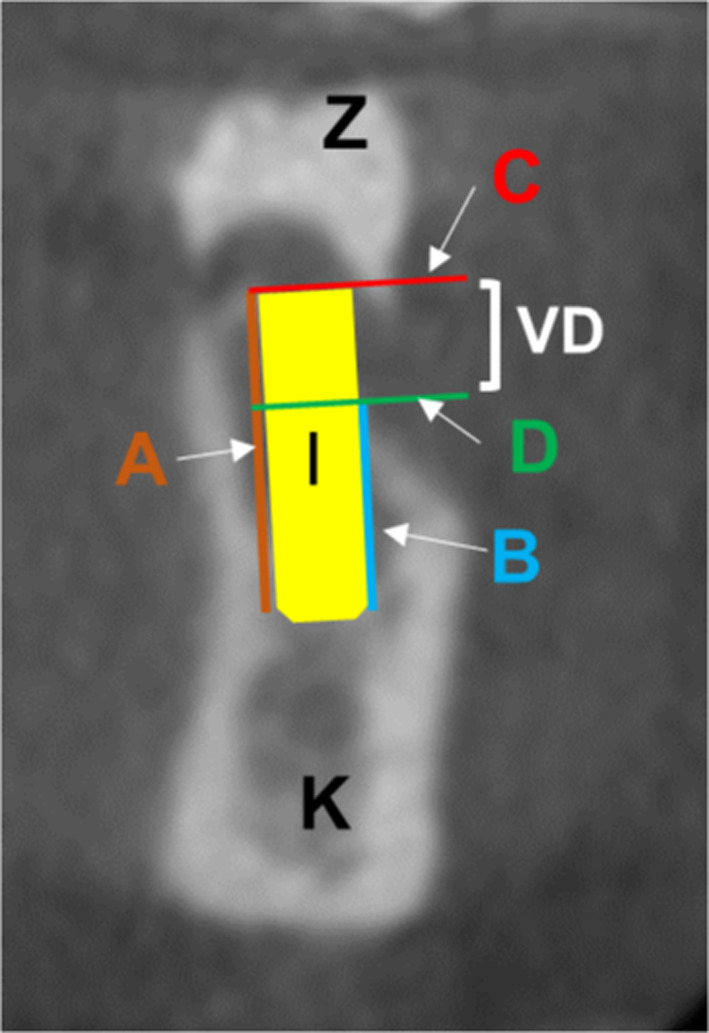
CBCT coronally slice: A and B lingual and buccal lines parallel to the implant axis, C and D lines perpendicular to A and B in order to determine the vertical distance (VD), K jawbone, I of the virtually planned implant

The variation in height (vertical distance) of the alveolar ridge between the lingual and buccal contour of the planned implant region was assessed in the edentulous jaw sections (Fig. 5). Radiopaque artificial teeth (Z) were placed in the planned implant position to determine the region. The virtual implant position (I) was planned following prosthetic aspects by an experienced dentist, who specialised in implantology and prosthetics. To this end, it was vital to cover the implant entirely with the lingual bone. A virtual line (A) was then drawn from the initial lingual contact point of the alveolar bone with the implant apex running parallel to the implant axis lingually. However, another line (B) was drawn from the initial buccal contact point of the alveolar bone the implant apex, running parallel to the implant axis buccally. Based on the contact points between the alveolar bone and the implant, the vertical distance (VD) was determined by two lines (C) and (D), drawn at right angles to lines (A) and (B).

The findings were fed into a mask developed at the Institute for Statistics (MediStat GmbH, Kronshagen, Germany) before being analysed by Mrs Ulrike von Hehn (Medistat GmbH, Kronshagen, Germany), using the SPSS Statistic 25 Software (IBM Corporation, Armonk, New York, USA). The results in this study have an explorative and descriptive character. All results therefore were expressed as absolute (quantitatively with mean and standard deviation) and incidence values (percentage).

## Results

Of 549 patients who received panoramic radiography and a CBCT during 2010–2017, 335 met the inclusion criteria. In the cohort study of 335 patients, 170 were females, and 165 were males. The average age of the female patients was 61.4 years, and that of the male patients was 58.1 years.

For both intra-observer reliability and inter-observer reliability, a kappa coefficient—according to Cohen—of 1.0 and a 95% confidence interval for kappa [0.92; 1.00] were calculated.

During the evaluation, two non-evaluable cases for the left side and one for the right side resulted in input errors. These cases (0.6% left and 0.3% right) were excluded from the evaluation; thus, the statistical analysis was based on 333 and 334 patients, respectively. Approximately 70% of the substantiating indications for obtaining a CBCT image were associated with implantology-related questions.

Tables 1 and 2 depict the detailed unusual contours in the edentulous jaw sections based on the panoramic radiography and CBCT observation in the maxilla. Here it can be seen that in most images no unusual jaw contour was recognisable, in a few images unusual jaw contours were visible in the CBCT, all of which were not recognisable in the panoramic radiography. From the analysis of the CBCT images, an unusual jaw contour was observed in 8 cases (2.4%) in the maxilla on the left, and in 10 cases (3%) in the maxilla on the right. However, no unusual jaw contours were detected in the panoramic radiography images (Tables 1, 2).

**Table 1:** Contingency table of unusual jaw contour of the left maxilla in per cent

			Panoramic radiography maxilla: unusual jaw contours visible on left side	Total
			No	
CBCT maxilla: unusual jaw contours visible on left side	No	Quantity	325	325
		% of the total	97.0%	97.0%
	Yes	Quantity	8	8
		% of the total	2.4%	2.4%
	System error	Quantity	2	2
		% of the total	0.6%	0.6%
Total		Quantity	335	335
		% of the total	100.0%	100.0%

**Table 2:** Contingency table of the unusual jaw contour of the right maxilla in per cent

			Panoramic radiography maxilla: unusual jaw contours visible on right side	Total
			No	
CBCT maxilla: unusual jaw contours visible on right side	No	Quantity	324	324
		% of the total	96.7%	96.7%
	Yes	Quantity	10	10
		% of the total	3.0%	3.0%
	System error	Quantity	1	1
		% of the total	0.3%	0.3%
Total		Quantity	335	335
		% of the total	100%	100%

Tables 3 and 4 indicate the identifiability of unusual jaw contours in the edentulous jaw sections in the mandible in panoramic radiography and CBCT images. From the CBCT images of the mandible, we detected an unusual jaw contour in 39 cases (12.1%) on the left side and 39 cases on the right side. In the panoramic radiograph images, an unusual jaw contour was identified in the left mandible. In the panoramic radiograph images, unusual jaw contours were diagnosed in three cases, not confirmed in CBCT images (Tables 3, 4). From the direct comparison of the corresponding images, these cases were presumably caused by translucency due to the mylohyoid line or oblique line, superimposed on the mandibular canal in panoramic radiography images. However, these could lead to misdiagnosis of the intervening translucent areas.

**Table 3:** Contingency table of unusual jaw contour of the left mandible in per cent

			Panoramic radiography mandible: unusual jaw contours visible on left side		Total
			No	Yes	
CBCT mandible: unusual jaw contours visible on left side	No	Count	278	3	281
		% of Total	86.3%	0.9%	87.3%
	Yes	Count	39	1	40
		% of Total	12.1%	0.3%	12.4%
	Not detectable / not in the field of view	Count	1	0	1
		% of Total	0.3%	0.0%	0.3%
Total		Count	318	4	322
		% of Total	98.8%	1.2%	100%

**Table 4:** Contingency table, in per cent of unusual jaw contour, of the right mandible

			Panoramic radiography mandible: unusual jaw contours visible on right side		Total
			No	Yes	
CBCT mandible: unusual jaw contours visible on right side	No	Count	279	3	282
		% of Total	86.6%	0.9%	87.6%
	Yes	Count	39	0	39
		% of Total	12.1%	0.0%	12.1%
	Not detectable/ not in field of view	Count	1	0	1
		% of Total	0.3%	0.0%	0.3%
Total		Count	319	3	322
		% of Total	99.1%	0.9%	100%

**Fig. 6:**
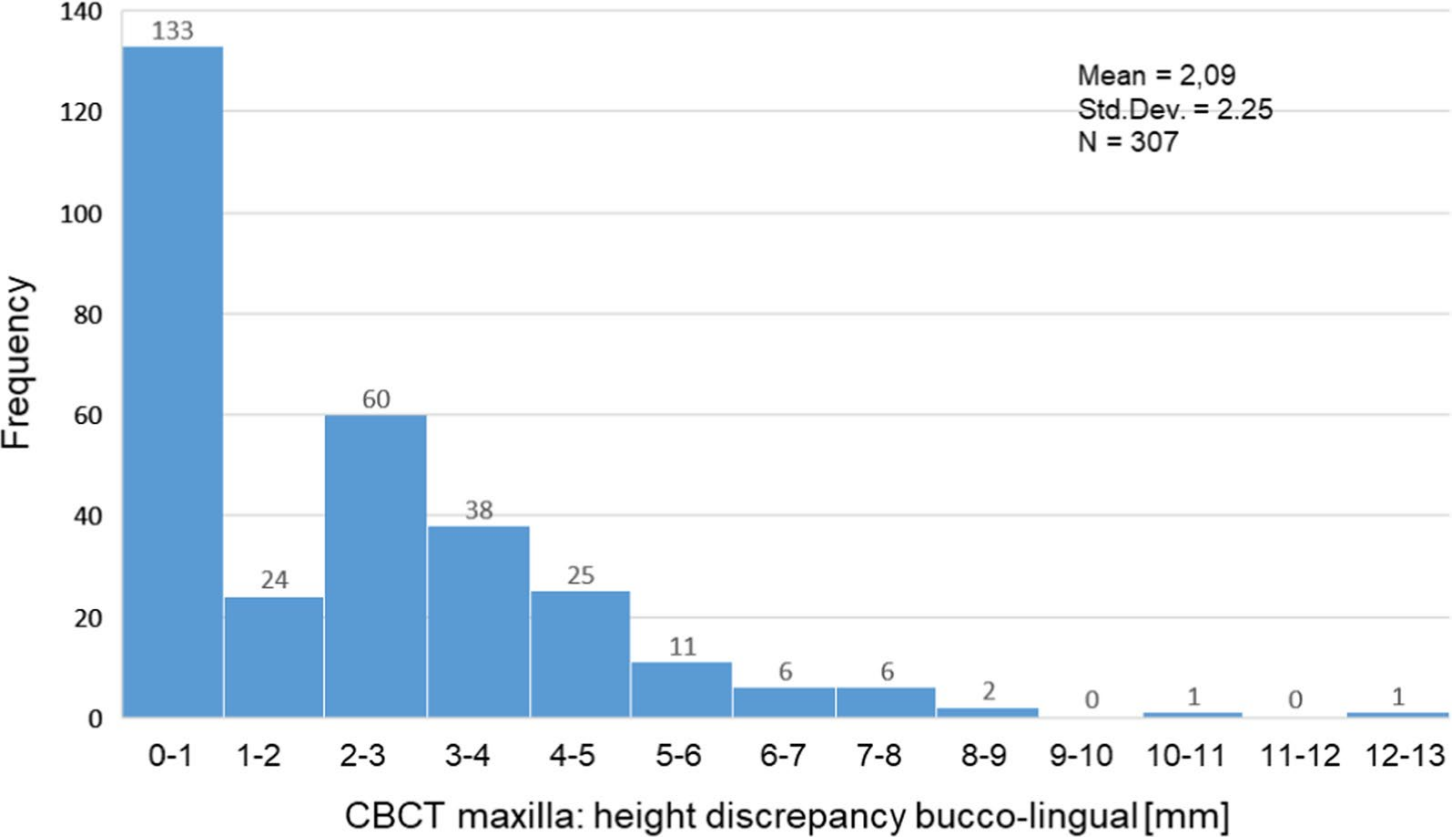
Frequency distribution of the height discrepancy of the maxilla calculated in 1-mm steps

**Fig. 7:**
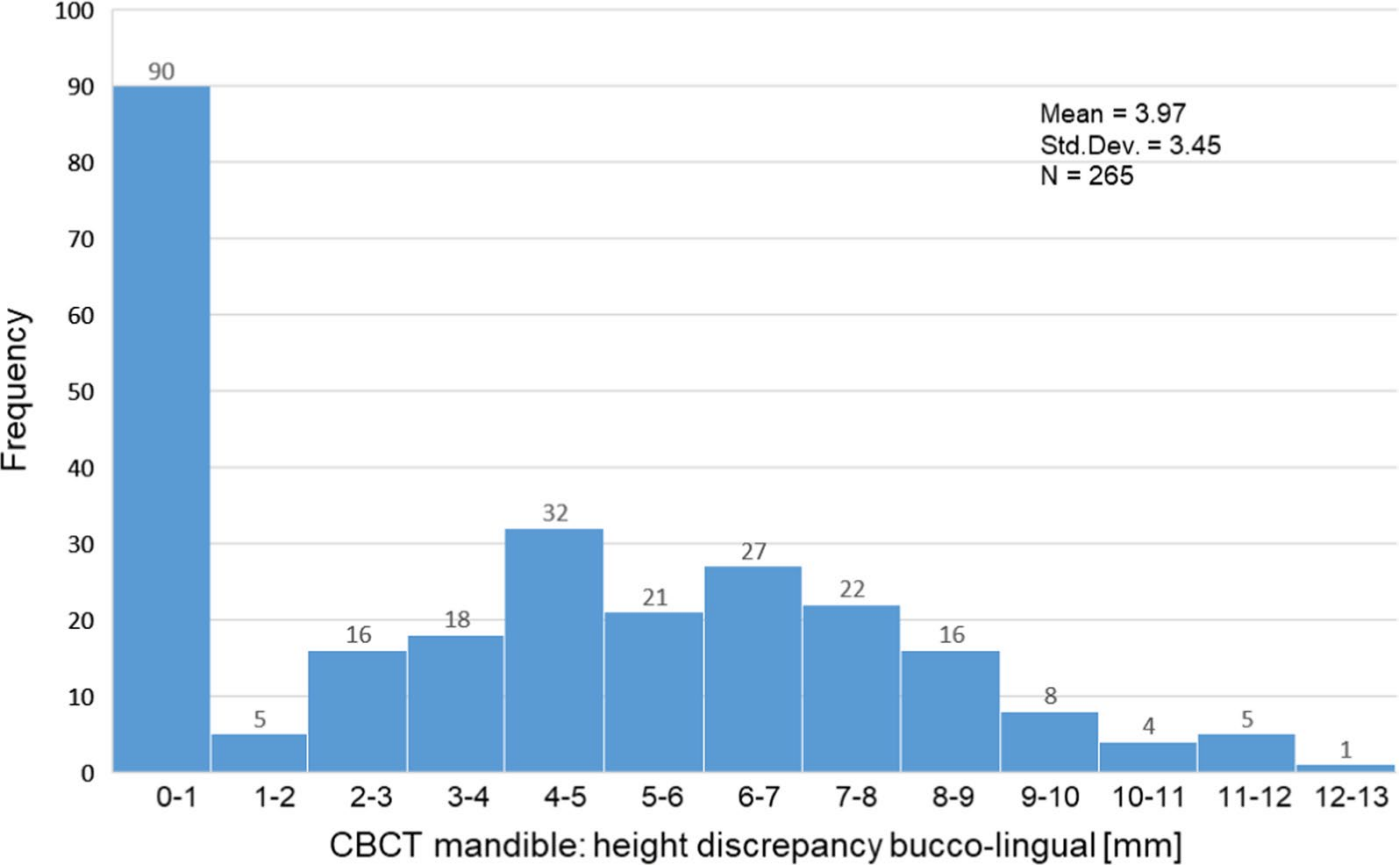
Frequency distribution of the height discrepancy of the mandible calculated in 1-mm steps

Figures 6 and 7 show the frequency distribution with regard to the buccolingual height discrepancy in the maxilla and in the mandible. In principle, smaller discrepancies in height were observed in the upper jaw. No or only slight vertical discrepancies (0–1 mm) were observed in 133 cases in the maxilla and in 90 cases in the mandible.

While the largest group in the upper jaw (*N* = 60) showed a height discrepancy of 2–3 mm, this was the case in the lower jaw (*N* = 32) with 4–5 mm. Larger discrepancies (5–12 mm) were observed much more frequently in the mandible.

Table 5 depicts the mean of the height difference for the maxilla and mandible, regarding the buccolingual aspect of the virtually planned implant axis. In the three-dimensional images, a height difference was detected in 307 cases in the maxilla and in 265 cases in the mandible. The vertical discrepancy was 2.09 ± 2.25 mm in the maxilla and 3.97 ± 3.45 mm in the mandible. Table 5 also shows the distribution of measurements in percentiles. The 25. Percentile showed a height discrepancy of 0.0 mm in both the mandible and the maxilla. In 50% of the measurements of the edentulous jaw sections of the maxilla, the discrepancy was up to 2.0 mm and up to 4.1 mm in the mandible. In 75% of the cases, the height discrepancy was up to 3.4 mm in the maxilla and up to 6.7 mm in the mandible.

**Table 5:** Buccolingual height discrepancy of edentulous region in mm

	*N*	Mean value	SD	Minimum	Maximum	Percentile		
						25.	50.	75.
CBCT maxilla	307	2.09	2.25	0.00	13.00	0.00	2.00	3.47
CBCT mandible	265	3.97	3.45	0.00	12.50	0.00	4.10	6.70

## Discussion

This study investigates whether unusual jaw contours of edentulous jaw sections, which are not visible in 2D images, could be detected in the transverse plane of CBCT images. Using the CBCT images, a further step is to measure the height difference of the alveolar ridge between lingual and buccal aspects in the planned implant position and edentulous jaw areas.

The hypothesis was that no difference was observed in assessing the contour in edentulous jaw sections or in measuring the buccolingual height in both 2D and 3D-dimensional images.

CBCT-based implant planning showed a vertical bone deficit on the buccal side of the planned implant in the maxilla of 307 patients and the mandible of 265 patients. The mean was 2.09 mm (SD ± 2.25 mm) in the maxilla with a maximum deficit of 13 mm, and 3.97 mm (SD ± 3.45 mm) in the mandible with a maximum deficit of 12.5 mm (Table 5). Slight height discrepancies (0–1 mm) were measured in 133 cases in the maxilla and 90 cases in the mandible. At 4.1 mm, the 50 percentile in the lower jaw was more than twice as high as in the upper jaw at 2.0 mm. Larger discrepancies (5–12 mm) were observed much more frequently in the mandible.

The findings indicate that an unusual jaw contour in the implant position is detected in 2.4% of cases on the left and 3% on the right in the maxilla. However, an unusual jaw contour in the implant position was detected in 12.1% of the left and 12% right in the mandible. However, these were not detected by panoramic radiography.

A total of 335 pairs of images were compiled and included for further analysis. In the preliminary stages, measurement integrity was verified by an expert. The measurements were repeated at 2-week intervals. Both intra-observer reliability of the principal examiner and inter-observer reliability between the principal examiner and expert were significantly high, resulting in a Cohen’s kappa coefficient of 1.0 and a 95% confidence interval for kappa [0.92; 1.00]. The high intra- and inter-observer reliability support the reliability of the results.

Since Mozzo et al.’s description of CBCT in 1998 [2], numerous scientific studies have addressed the question of the CBCT necessity before surgical procedures. More specifically, the necessity for obtaining a CBCT scan for implant surgery has remained controversial [1, 11].

Few studies [24–27] compare panoramic radiography and CBCT for detecting height differences between the lingual and buccal ridges of edentulous jaw sections. Wolff et al. [24] conducted a comparative study of panoramic radiography and CBCT scans in 253 patients to ascertain whether CBCT scans affected the planning of dental implants. While CBCT images yielded more useful information, they did not influence the operative plan of this study, based on a pre-existing panoramic radiography image. Nevertheless, no direct comparison was made between panoramic radiography and CBCT. The results showed a bone deficit on the buccal side of these jaw sections in CBCT. Before the planned implantation, additional information provided by the CBCT can play an important role in the decision of the surgeon and the patient. Particularly, the CBCT seems to leave little room for interpretation of the findings, which is substantiated by Malina-Altzinger et al. [25]. In their comparative study, they concluded that certain findings in panoramic radiography could be based on the assessment of the examiner. Compared with panoramic radiography, the authors conclude that CBCT allows examiner-independent assessment of specific findings, relevant to planned subsequent surgical interventions.

Deeb et al. [26], Dagassan-Berndt et al. [27] and Guerrero et al. [22] show similar results. The latter study also revealed that CBCT-based preoperative implant planning facilitated a significantly higher prognosis regarding augmentation than panoramic radiography-based planning.

Our findings are consistent with those of the above-cited studies [22, 26, 27]. In cases with more extensive bone deficits, CBCT-based implant planning has a substantial additional value for diagnostics. Additional information can provide a positive outcome for assessing implantation feasibility, augmentation and evaluation of costs and risks, which could offer possible forensic evidence.

Other aspects examined in the current study were unusual jaw contours, such as funnel-shaped or pronounced retraction of the edentulous sections of the jaw. Moreover, corresponding panoramic radiographs and CBCT images of edentulous sections of the jaw were compared. This information is vital for planning before implantation to minimise the risk associated with damaging adjacent structures.

Rajput et al. [28] used CBCT to assess the lingual concavities in the submandibular fossa in patients with missing posterior teeth, requiring dental implants. In 62% of the patients, a concavity depth of 2–3 mm was present; whereas in 15% of the cases, a concavity depth greater than 3 mm was determined. These authors conclude that CBCT remains the most effective imaging technique for providing sufficient information when planning posterior implant surgery. Another study used a design similar to that of Nickenig et al. [17] and showed that the undercut of edentulous mandibles was detected in the molar region of 68% of the patients.

Our results showed that an unusual jaw contour in the planned implant position could be detected by CBCT in 2.4% of cases on the left and 3% on the right in the maxilla. The results also show 12.1% on the left and 12.1% on the right in the mandible, otherwise not detected by panoramic radiography, which may be significantly lower than the values presented by the abovementioned studies. The discrepancies could appear because we did not measure the depth of the concavity. In our study, it was important to determine whether implant planning in the edentulous section of the jaw was possible without causing perforation. Thus, minor undercuts were not accounted for. The small number of undetected unusual jaw contours should not conceal the serious complications during implant surgery. Perforation of the lingual wall while preparing the implant bed can cause profuse bleeding, concomitant swelling and infection in the parapharyngeal space [10, 29]. Precise information on the anatomical structures can considerably mitigate perforation risks [30]. Comparable results were published by Shelley et al. [31]. Eight clinicians performed implant planning in the anterior mandible using panoramic radiography and CBCT. In challenging cases, narrower implants were selected when CBCT was available, presenting a little risk of lingual cortical perforations. In cases with known unusual jaw contours, another strategy was to use short implants. This might reduce the risk of perforations or the requirement for augmentation. The findings of a recent study showed that short implants exhibit a high success rate, even after 5 years [32]. This minimises the risk of perforation and the requirement for augmentation.

Dau et al. [19] took different positions by comparing the subjective quality assessment of panoramic radiography and CBCT for the planning of dental implants by dentists with varying educational backgrounds. Their results showed significant subjective advantages for additional CBCT for planning dental implant procedures, particularly in the anterior and posterior maxilla. Above all, participants with only basic training requested CBCT more frequently. Other studies show that subjective factors, such as practitioner opinion or experience, are instrumental in assessing the viability of CBCT [22, 26].

Thus, our results indicate that useful information can be obtained by performing a CBCT before implant planning. This is crucial when assessing aspects such as risks, costs, duration of treatment or even forensic issues for the surgeon and patient. Consequently, our hypothesis may be rejected, which shows no difference is identified when assessing the contour in edentulous jaw sections or measuring the buccolingual height in both 2D and 3D images.

## Conclusion

Our final evaluation shows that CBCT is more effective than panoramic radiography because CBCT is 3D, uses a more complex method, yields more useful information and provides superimposition-free information. Yet, panoramic radiography cannot be discarded, notably in simple cases of dentistry. Regarding surgical planning in implantology, the CBCT scan provides useful information and helps to avoid complications in the perioperative planning phase. The anatomical variations and deviations from the norm can be considered in diagnosis and treatment planning. In recent years, CBCT has been increasingly practicable for surgical planning. Compared with panoramic radiography, which used ionising radiation, CBCT should be used because its potential benefits to the patient outweigh the risks.

Further studies are essential for establishing CBCT in implant planning, surgery and prosthetics, and forensics planning.

## Data Availability

The original datasets analysed in the current study are available from Dr. Ali Reza Ketabi on reasonable request.

## Abbreviations

CBCT: Cone-beam computed tomography
PR: Panoramic radiography
sec: Second
kV: Kilo volt
mA: Milli ampere
mm: Millimetre
cm: Centimetre
SD: Standard deviation
